# Valorization of Banana Peel Waste into Advanced Adsorbent Beads for the Removal of Emerging Pollutants from Wastewater

**DOI:** 10.3390/ma18051084

**Published:** 2025-02-28

**Authors:** Olivia Boyle, Bo Xiao, Chirangano Mangwandi

**Affiliations:** School of Chemistry & Chemical Engineering, Queen’s University Belfast, Belfast BT7 1NN, UKb.xiao@qub.ac.uk (B.X.)

**Keywords:** adsorption, emerging pollutants, tetracycline, chromium, sodium alginate, magnetic, encapsulation

## Abstract

This study addresses environmental concerns by utilizing banana peel waste to develop innovative adsorbent materials for wastewater treatment, aligning with circular economy principles. Spherical beads were synthesized from sodium alginate mixed with various banana peel-based materials, including pure powder (PBP), activated carbon (AC), and magnetic activated carbon (MAC). These beads were evaluated for their efficiency in removing tetracycline (TC) and hexavalent chromium (Cr(VI)) as model pollutants representing antibiotics and heavy metals, respectively. Characterization of the beads revealed functional groups and thermal stability conducive to effective adsorption. Adsorption trials demonstrated that MAC beads achieved the highest removal efficiencies, up to 92% for TC and 79% for Cr(VI). The adsorption process followed pseudo-second-order kinetics and Langmuir isotherms. Remarkably, the beads retained a significant adsorption capacity across reuse cycles, indicating their regenerative potential. Comparisons with other adsorbents highlight the competitive performance of these banana peel-based materials. The results emphasize the potential of banana peel-derived adsorbents as cost-effective, sustainable solutions for mitigating emerging pollutants in water systems, promoting waste valorization and environmental protection. The research demonstrates a novel approach to sequential adsorption without intermediate regeneration, showing that the beads can effectively remove both tetracycline and chromium (VI) in successive cycles. This finding is particularly significant because it reveals that the presence of previously adsorbed chromium actually enhanced the beads’ capacity for tetracycline removal in the second cycle, suggesting a synergistic effect that had not been previously reported in the literature. These innovations contribute meaningfully to both waste valorization and water treatment technologies, offering new insights into the development of multi-functional adsorbents from agricultural waste materials.

## 1. Introduction

Water pollution significantly impacts the availability of safe, clean water, posing a major health risk worldwide. Contaminated water sources can lead to diseases such as cholera, dysentery, and typhoid, affecting millions of people [1,2]. The lack of access to clean water exacerbates malnutrition and childhood stunting, particularly in low-income countries [2].

Wastewater treatment and the circular economy offer promising solutions to this challenge. By treating wastewater, we can remove harmful contaminants and safely reuse water, reducing strain on freshwater resources [3]. The circular economy promotes efficient water use through waste avoidance, recycling, and quality retention, ensuring environmental protection and conservation [4,5]. This approach extends to utilizing waste materials as resources for creating useful products, minimizing pollution while maximizing resource efficiency. Heavy metals such as chromium, cadmium, lead, and antibiotics are some of the pollutants that are of major concern. This study will use chromium and tetracycline as model pollutants for heavy metals and antibiotics, respectively.

Chromium (VI), also known as hexavalent chromium, is a highly toxic form of chromium that exists in the +6 oxidation state. It is primarily produced through industrial processes such as electroplating, leather tanning, and the manufacturing of pigments and dyes [6]. Chromium (VI) pollution occurs when these compounds are released into the air, water, or soil, often through industrial waste and emissions. The health impacts of chromium (VI) exposure are severe, including respiratory problems, lung cancer, kidney damage, and skin ulcers [7]. Environmentally, it can contaminate water sources and harm aquatic life. Current removal methods include chemical reduction, precipitation, and advanced oxidation processes. In the industrial context, chromium (VI) is significant due to its widespread use in various applications, despite its toxicity and the stringent regulations governing its use. The removal of chromium (VI) from wastewater is crucial due to its high toxicity and carcinogenic properties [8]. Found in industrial effluents from leather tanning, electroplating, and textile manufacturing [9], chromium (VI) exposure can lead to severe health issues, including respiratory problems, skin ulcers, and increased lung cancer risk. It also poses significant environmental risks, contaminating water sources and affecting aquatic life [9].

Water pollution by antibiotics, particularly tetracycline, is a growing environmental concern. Tetracycline, widely used in human and veterinary medicine, often enters aquatic environments through various sources such as hospital effluents, agricultural runoff, and improper disposal of pharmaceuticals [10,11]. Its prevalence in water bodies is significant, with concentrations detected in surface waters, sediments, and even drinking water [12]. Tetracycline is known for its environmental persistence due to its resistance to natural degradation processes, leading to prolonged presence in ecosystems [10]. This persistence poses serious health implications, including the disruption of microbial communities, the promotion of antibiotic resistance, and potential toxicity to aquatic organisms [12,13]. Additionally, the removal of tetracycline from water is challenging due to its complex chemical structure, which requires advanced treatment methods such as adsorption, advanced oxidation processes, and biological treatments [14]. Addressing tetracycline pollution is crucial to mitigate its environmental and health impacts. The removal of antibiotics, particularly tetracycline, from wastewater is essential due to their environmental persistence and potential health risks [15].

Current technologies for removing heavy metals and antibiotics from wastewater include a variety of advanced methods. Ion exchange processes are also effective, particularly for heavy metals, using resins to exchange ions with contaminants in the water [16]. Membrane filtration techniques, such as reverse osmosis and nanofiltration, are highly effective for both heavy metals and antibiotics, though they can be costly and require significant energy [17]. Advanced oxidation processes (AOPs), including photocatalysis and ozonation, are particularly effective for degrading antibiotics and other organic contaminants [17]. Research has also shown that in carbon-based materials, presences of persistent free radicals (PFRs) can also contribute to the removal of pollutants through oxidative degradation reactions [18,19,20]. Electrochemical methods offer another approach, using electric currents to remove contaminants through processes like electrocoagulation and electrooxidation [16]. Biological treatments, such as constructed wetlands and microbial degradation, are gaining attention for their sustainability and ability to handle a range of contaminants [21]. Adsorption is widely used due to its efficiency and cost-effectiveness, employing materials like activated carbon, biochar, and novel adsorbents enhanced with nanotechnology [16].

These technologies often face challenges related to scalability, cost, and the need for specialized infrastructure, but ongoing research continues to improve their efficiency and feasibility [16,17,21]. Among these technologies, adsorption is the most used due to its simplicity and low cost. The operational cost of adsorption can further be reduced by using a highly efficient low-cost adsorbent. The current research takes a circular-economy approach to developing low-cost adsorbents from waste.

Food waste valorization, particularly from banana-based materials, presents sustainable solutions to address environmental challenges. Banana production generates substantial agricultural waste, with peels constituting approximately 35–40% of the total fruit weight. Global banana production exceeds 120 million tonnes annually, resulting in roughly 42 million tonnes of peel waste. This waste typically ends up in landfills where it contributes to greenhouse gas emissions through decomposition. Banana waste can be utilized in various innovative ways, including the production of bioplastics by leveraging its rich content of cellulose, hemicellulose, and lignin [22]. Additionally, banana waste can be used for bioethanol production through the fermentation of its sugars and fibres [23]. In agriculture, banana waste serves as an effective organic fertilizer and compost material, enriching the soil naturally [24]. Furthermore, it can be used as animal feed, providing a cost-effective, nutrient-rich dietary supplement for livestock [25]. Finally, banana waste finds applications in the pharmaceutical and cosmetic industries, where its antioxidants and bioactive compounds are highly valued. These diverse applications highlight the potential of banana waste to contribute to a more sustainable future [26].

Banana peels and their derivatives have demonstrated significant potential as adsorbents for water treatment. Their natural composition, rich in lignin and cellulose, contributes to their high adsorption behaviour [27]. These materials exhibit pH sensitivity, with optimal removal of cationic pollutants occurring at pH levels between 5.0 and 7.0, and anionic pollutants at pH levels between 2.0 and 4.0 [28]. The pollutant removal capacity of banana peels is impressive, effectively eliminating heavy metals such as chromium (Cr), copper (Cu), lead (Pb), and zinc (Zn) [29,30]. They have also been successfully applied in the treatment of textile industry wastewater [31], removing dyes, and have shown efficacy in eliminating pharmaceuticals like amoxicillin and carbamazepine [32]. Additionally, banana peels have proven to be highly efficient in removing antibiotics such as ciprofloxacin and doxycycline [33,34]. Recent studies have reported removal efficiencies of up to 98% for heavy metal ions and 98.93% for organic and inorganic pollutants. These adsorbents offer cost-effective and sustainable solutions, with the potential for regeneration and reuse [33,35].

This study advances the field through the development of spherical beads incorporating various forms of banana peel-based particles in sodium alginate. These composite materials, particularly the magnetic activated carbon beads (MAC@NaAlg), represent a novel approach to waste valorization and water treatment. The research evaluates their effectiveness in removing tetracycline and hexavalent chromium as model pollutants for antibiotics and heavy metals, respectively.

## 2. Experimental Methods

### 2.1. Materials

Otho-phosphoric acid (H_3_PO_4_), Potassium dichromate (K_2_Cr_2_O_7_), iron (III) nitrate nonahydrate (Fe(NO_3_)_3_·9H_2_O), iron (II) sulphate heptahydrate (FeSO_4_·7H_2_O), sodium hydroxide(NaOH), hydrochloric acid (HCl), and calcium chloride (CaCl) were all purchased from Sigma Aldrich (Gillingham, UK). Sodium alginate (NaC_6_H_7_O_6_) was purchased from Fisher Scientific UK Ltd. (Loughborough, UK).

### 2.2. Production of Biochar

In preparation for the procedure, the banana peel was washed with deionized water and dried before undergoing size reduction by blending, achieving an average particle size of 125 µm. In brief, the samples were pre-treated by soaking and saturating with orthophosphoric acid (H_3_PO_4_) at an 85% concentration, using an acid-to-sample ratio of 1:1. The samples were then mixed with a magnetic stirrer for 1 h to ensure homogeneity.

Subsequently, the samples underwent a two-step heating process. First, they were subjected to a temperature of 150 °C for 2 h, followed by a further 2 h in a furnace under a flow of nitrogen at 400 °C. After cooling, the samples were washed with water and dried for 24 h.

### 2.3. Preparation of Magnetic Biochar

The biochar was then activated using iron (III) nitrate nonahydrate and iron(II) sulphate heptahydrate. Specifically, 1 g of biochar was suspended in a 500 mL solution containing 3.5 g of Fe(NO_3_)_3_ and 1.3 g of FeSO_4_·7H_2_O. The suspension was sonicated at 40 W for 10 min. To precipitate the iron oxide, an aqueous solution of sodium hydroxide (NaOH; 0.1 mol) was added drop-wise until the pH reached between 11 and 12. The mixture was then vigorously stirred at 50 °C for 1 h. Subsequently, the precipitate was separated from the aqueous dispersion by filtration and washed repeatedly with double-distilled water until the pH was neutral. The magnetic activated carbon was then dried and sealed in a container for later use.

### 2.4. Preparation of Adsorbent Beads

The beads were produced from four different materials: pure sodium alginate powder, 1:1 blends of sodium alginate powder with powdered banana peel (PBP), banana peel activated powder (AC), and magnetic activated carbon (MAC). These materials were, respectively, named NaAlg, PBP@NaAlg, AC@NaAlg, and MAC@NaAlg. Spherical adsorbent beads were synthesized using the following gelation procedure:

1. Mixing: First, 4 g of sodium alginate powder was thoroughly mixed with 4 g of the various materials (including unmodified banana peel, activated carbon, and magnetic activated carbon) in a weigh boat. The resulting mixed powder was slowly added to 400 mL of deionized water while continuously stirring. The mixture was left in the beaker overnight to ensure homogeneity.
2. Calcium Chloride Solution: Next, a 2 wt% solution of calcium chloride was prepared by dissolving calcium chloride in 400 mL of deionized water. This solution was maintained at room temperature on a hot plate.
3. Drop-Wise Addition: While the calcium chloride solution was magnetically stirred, the various material solutions were passed through a pear-shaped separating funnel and added drop-wise to the beaker.
4. Gelation Process: Following the gelation process, the beads formed were subjected to gentle magnetic stirring at 350 rpm for 24 h in their mother liquor.
5. Harvesting and Drying: Finally, the beads were harvested by filtering the calcium chloride solution and washing them several times with deionized water. Subsequently, the beads were dried at room temperature in a fume cupboard.

### 2.5. Characterization

Fourier Transform Infrared (FTIR) Spectroscopy was employed to identify the functional groups of each sample before and after adsorption, within the range of 4000–600 cm^−1^. Additionally, X-ray diffraction (XRD) tests were performed using a Philips PANalytical X’pert Pro diffractometer (Malvern Instruments, Worcestershire, UK) to determine the crystalline and amorphous characteristics of the best adsorbent sample. Surface area and porosity information for each sample were obtained through Brunauer–Emmett–Teller (BET) analysis at 77 K, using a Micromeritics Tristar 3020 instrument (Micrometrics Instrument Corporation, Norcross, GA, USA) with a nitrogen gas flow. The thermal stability of the samples was assessed using thermogravimetric analysis (TGA) under nitrogen gas flow, starting at room temperature and heating to 800 °C at a rate of 5 K min^−1^.

### 2.6. Adsorption Trials

The different samples of adsorbent beads were tested for the adsorption of chromium and tetracycline. Each sample was put in contact with aqueous solutions of either chromium or tetracycline with an initial concentration of 150 ppm. The residual concentration of the solutions of chromium and tetracycline were measured using UV spectrophotometry at wavelengths of 540 and 357 nm, respectively.

The concentration of the pollutant loaded onto the adsorbent was calculated from the initial concentration, $C_{i}$, the residual concentration, $C_{e}$, the mass of adsorbent sample m and the volume of solution V using (1):

(1) $$q_{e}=\frac{V\left(C_{i}-C_{e}\right)}{m}$$

The removal efficiency of the adsorbent was calculated from

(2) $$R_{eff}=\frac{100\left(C_{i}-C_{e}\right)}{C_{i}}$$

The symbols in (2) are as defined previously.

From the adsorption isotherm study, the adsorbents were put in contact with solutions of the pollutant in various concentrations ranging from 25 to 150 ppm.

### 2.7. Initial Concentration Effect and Isotherm Modelling

Solutions of chromium with concentrations ranging from 25 to 150 ppm (in 25 ppm increments) were prepared from the stock solutions. The pHs of the solutions were adjusted to pH 2 using either HCL or NaOH. Amounts of 20 mL of these solutions were transferred into 6 separate glass vials and 50 mg of the adsorbent PBP beads. This was repeated for the other types of beads. The vials were placed on the shaker at 100 rpm for agitation for a contact time of 24 h. At the end of the contact time, the beads were filtered from the solution and dried for storage and future characterization. Samples were taken from the filtrate and diluted for concentration determination by UV-Vis spectroscopy using the procedure previously reported [29]. A measure of 6 mL of each sample was mixed with 2 mL of colour development reagent. UV analysis was performed at a wavelength of 540 nm. The same procedure was repeated for the tetracycline, and the only differences in the procedure were that no pH adjustment of the solution was required and also no colour development reagent was required prior to UV analysis. The UV analysis was performed at a wavelength of 357 nm. Equation (1) was used to calculate $q_{e}$ values.

(i) Isotherm Modelling

Isotherm models play a critical role in understanding the interactions between adsorbates and adsorbents. They provide valuable insights into the adsorption capacity of the material, helping to identify the most efficient and cost-effective adsorbent for specific applications. These models also enable the prediction of adsorption performance under varying conditions, which is essential for scaling-up laboratory findings to industrial processes.

The data were analyzed using several isotherm models, including Freundlich (3) and Langmuir (4).

(3) $$q_{e}=k_{F}C_{e}^{\frac{1}{n}}$$

(4) $$q_{e}=\frac{q_{max}k_{L}C_{e}}{1+k_{L}C_{e}}$$

In Equations (3) and (4) where *C_e_* (mmol/L) is the equilibrium concentration of the adsorbate solution, *q_m_* (mmol/g) is the maximum amount of adsorbate removed by the adsorbent, *q_e_* is the removal capacity at equilibrium (mmol/g), *K_L_* and *K_F_* are the Langmuir constants related to the interaction bonding energies and the Freundlich affinity coefficient, respectively, and n is a correction factor in the Freundlich equation.

The model parameters were obtained from non-linear regression fits of the isotherm models to the experiment data using custom MATLAB code (MATLAB Version 2024b).

### 2.8. Reuse of the Beads

After the adsorption tests, the beads were recovered from the solution through filtration. They were then dried in an oven at 60 °C and stored in sealed jars until further use. The beads originally used for tetracycline adsorption were subsequently evaluated for Cr(VI) adsorption, and vice versa.

## 3. Results

### 3.1. Characterization Results

(i) Ultimate analysis

A CHNS elemental analysis was conducted on powdered banana peel, AC@NaAlg, and MAC@NaAlg. The results, presented in Table 1, show the total percentages of carbon, hydrogen, nitrogen, and sulphur in the samples. The analysis reveals a decrease in carbon content from the initial material, ‘BPP’, which had a carbon content of 39.38%, to AC@NaAlg with 24.71%, and finally to the synthesized material MAC@NaAlg, which had the lowest carbon content at 19.89%.

This reduction in carbon content from the banana peel to the activated carbon can be attributed to the activation process. The high temperatures and the use of the activating agent H_3_PO_4_ remove non-carbon components such as ash and various volatile organic compounds. The carbon content of the banana peel powder aligns with values reported in the literature by [36], which indicate a carbon content of 47.5%. The hydrogen, nitrogen, and sulphur contents were like those reported in Table 1.

(ii) Analysis of functional groups through FTIR

**Figure 1:**
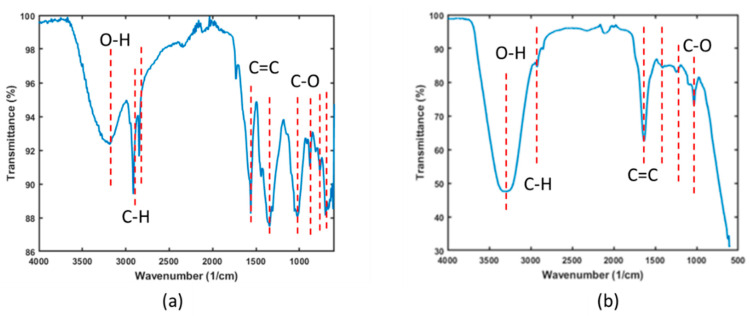
FTIR spectra for (**a**) banana peel powder and (**b**) magnetic activated carbon beads.

The FTIR spectra presented in Figure 1 illustrate the functional groups present in both the banana peel powder and the final product, magnetic activated carbon beads. The spectra reveal hydroxyl and carboxyl groups in the banana peel sample, with bands at 3350 cm^−1^ and 2800 cm^−1^, respectively.

The band around 1680 cm^−1^ is attributed to the asymmetric C=C stretch from the ether group [37]. In the BPP sample, the band at approximately 1010 cm^−1^ indicates the presence of C-O, C-OH, and C-C stretching, corresponding to the hemicellulose, cellulose, and lignin in the banana peel powder [38]. Weak bands observed at around 800 to 600 cm^−1^ are attributable to amine groups.

(iii) Thermogravimetric analysis

**Figure 2:**
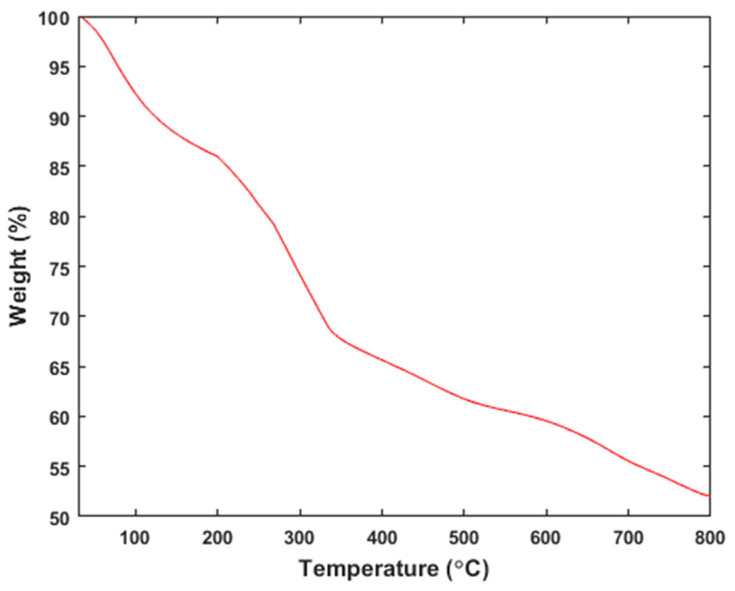
TGA thermograph of the MAC@NaAlg beads.

Figure 2 presents the Thermogravimetric (TG) curve for the magnetic activated carbon (MAC) beads, which were obtained at a heating rate of 10 °C/min. As depicted in Figure 2, a decrease in sample weight of approximately 10% is observed when the temperature is increased from room temperature to 120 °C. This reduction in sample weight is primarily due to the evaporation of water from the beads.

Upon the further heating of the sample, a significant weight loss of around 20% occurs between 200 and 320 °C, followed by another decrease in the range of 330 to 600 °C. The weight loss in the first range is indicative of the decomposition of cellulose and hemicellulose in the beads, which originate from the banana peel powder used in the production of the beads [39].

The subsequent weight loss at higher temperatures can be attributed to the decomposition of other components, such as lignin [40]. The results demonstrate that the MAC@NaAlg beads exhibit thermal stability up to approximately 180 °C.

### 3.2. Comparison of Adsorption Performance

(i) Removal of TC with the virgin powder and the beads

**Figure 3:**
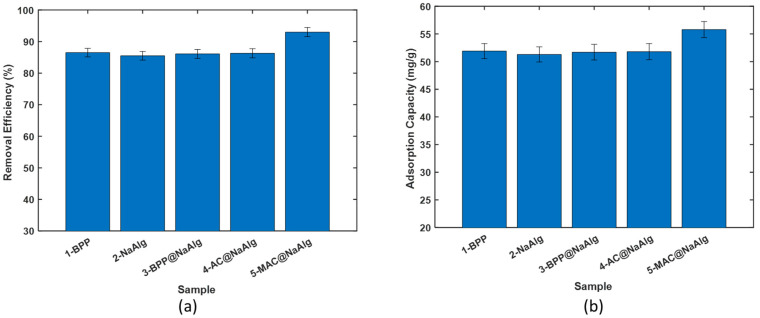
Comparison of the (**a**) TC removal efficiency and (**b**) TC adsorption capacity of the different materials.

Figure 3a presents the comparison of the removal efficiency of various bead types in treating aqueous solutions of tetracycline. The beads demonstrated removal efficiency ranging from 85 to 92%. The highest efficiency was achieved using magnetic activated beads as the adsorbent. Interestingly, the other four bead types achieved a removal efficiency of approximately 86%, with no significant difference among them. These data underscore the benefits of incorporating magnetic nanoparticles into the activated carbon used to create the MAC@NaAlg beads.

Remarkably, despite differences in particle size, the removal efficiency of banana peel powder was comparable to that of NaAlg. Figure 3b displays the amount of tetracycline captured by the beads during the removal process, as calculated using (1). The trends observed here mirror those seen in the removal efficiency.

The adsorption capacities of BPP, NaAlg, BPP@NaAlg, and AC@NaAlg were all around 52 mg/g, with the differences between them being statistically insignificant. However, the removal capacity of MAC@NaAlg was significantly higher than that of the other four materials.

(ii) Removal of Cr(VI) with the virgin beads

**Figure 4:**
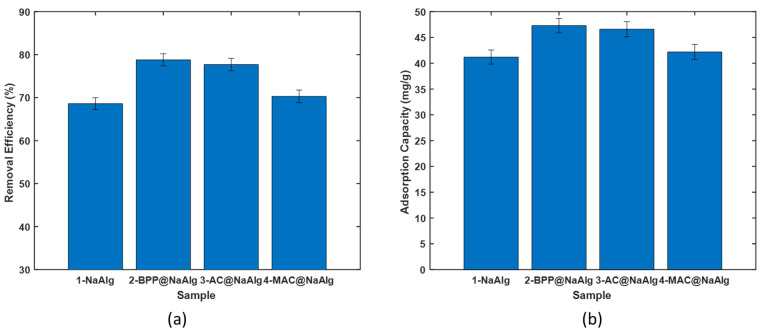
Comparison of the (**a**) Cr (VI) removal efficiency and (**b**) Cr(VI) adsorption capacity of the different materials.

The comparison of the removal efficiency among various bead types for treating aqueous solutions of chromium (VI) is presented in Figure 4a. The beads exhibited a removal efficiency ranging from 68% to 79%. Notably, the removal efficiency was significantly higher for beads encapsulating other materials than for pure sodium alginate beads. The highest efficiency was achieved using magnetic activated beads (MAC) as the adsorbent. Interestingly, there were no significant differences in removal capacities between BPP@NaAlg, BPP@NaAlg, and AC@NaAlg beads.

Figure 4b illustrates the amount of chromium (VI) captured by the beads during the removal process, reflecting trends similar to the removal efficiency. The adsorption capacities of NaAlg, BPP@NaAlg, and AC@NaAlg fell within the range of 41 to 47 mg/g. Pure sodium alginate beads exhibited the lowest adsorption capacity, approximately 41 mg/L.

### 3.3. Effect of Initial Concentration Study

**Figure 5:**
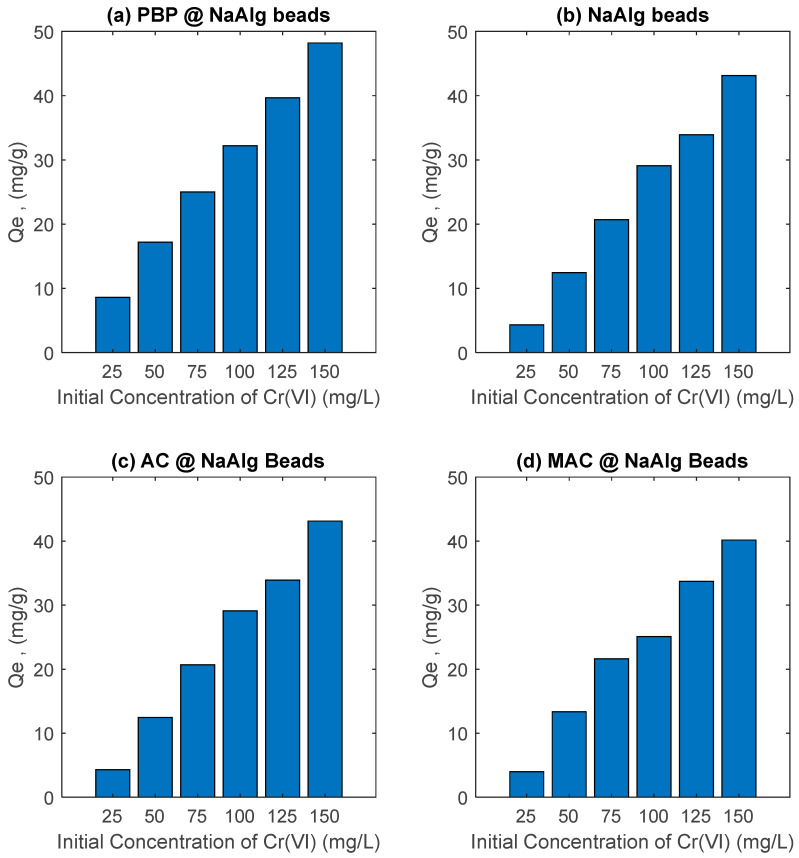
Effect of initial concentration of Cr(VI) on its removal by various beads. Study performed at room temperature with dosage of adsorbent beads of 2.5 g/L.

Figure 5 illustrates the effect of the initial Cr(VI) concentration on the adsorption capacity ($Q_{e}$) of various banana peel-based adsorbent beads, including sodium alginate beads (NaAlg), activated carbon-loaded sodium alginate beads (AC@NaAlg), magnetic activated carbon beads (MAC@NaAlg), and powdered banana peel beads (PBP@NaAlg). Across all adsorbents, the adsorption capacity increased as the initial Cr(VI) concentration increased from 25 to 150 mg/L. This trend is expected because higher initial Cr(VI) concentrations provide a greater driving force for mass transfer, enhancing the adsorption onto the surface of the beads.

Among the tested materials, AC@NaAlg and MAC@NaAlg exhibited the highest adsorption capacities, suggesting that the incorporation of activated carbon, especially in its magnetic form, significantly enhances adsorption performance. This improvement can be attributed to the high surface area, porosity, and functional groups of activated carbon, which facilitate Cr(VI) uptake through both physisorption and chemisorption mechanisms [41]. Similarly, PBP@NaAlg demonstrated a relatively high adsorption performance, which may be due to the functional groups present in banana peel powder, such as hydroxyl and carboxyl groups, that interact with Cr(VI) ions [42]. In comparison, the pure sodium alginate beads (NaAlg) showed the lowest adsorption capacity, highlighting the limited adsorption potential of alginate alone. This suggests that incorporating fillers like activated carbon or banana peel powder into the alginate matrix significantly improves the adsorption efficiency.

The observed adsorption capacities of AC@NaAlg and MAC@NaAlg align well with previous studies. For instance, magnetic activated carbon composites have been shown to exhibit high Cr(VI) adsorption capacities due to their high surface area and magnetic properties, which facilitate adsorption and subsequent separation [43,44]. Additionally, agricultural waste-based adsorbents, such as modified biochar and banana peel-derived adsorbents, have demonstrated adsorption capacities ranging from 20 to 50 mg/g under similar conditions, depending on the preparation method and experimental setup [45,46]. The results in the current study suggest that MAC@NaAlg and AC@NaAlg are promising adsorbents for Cr(VI) removal, performing comparably or even superiorly to conventional and agricultural waste-based materials reported in the literature.

**Figure 6:**
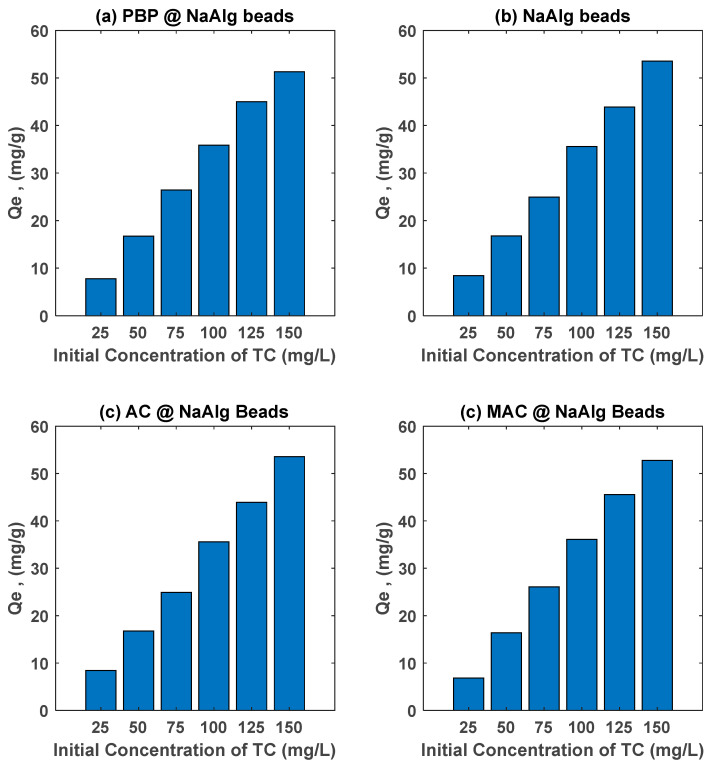
Effect of initial concentration of tetracycline on its removal by various beads. Study performed at room temperature with dosage of adsorbent beads of 2.5 g/L.

Figure 6 illustrates the effect of initial tetracycline (TC) concentration on the adsorption capacity (Qe) of various banana peel-based adsorbent beads, including sodium alginate beads (NaAlg), activated carbon-loaded sodium alginate beads (AC@NaAlg), magnetic activated carbon beads (MAC@NaAlg), and powdered banana peel beads (PBP@NaAlg). The adsorption capacity of all materials increased with the increasing initial TC concentration (25–150 mg/L) after 24 h of contact time with a fixed bead dosage of 2.5 g/L. This behaviour can be attributed to the greater driving force for mass transfer at higher initial concentrations, which enhances the interaction between TC molecules and the adsorbent surfaces.

Among the tested materials, AC@NaAlg and MAC@NaAlg exhibited the highest adsorption capacities, demonstrating the superior adsorption performance provided by the presence of activated carbon. The high surface area and porosity of activated carbon facilitate the effective adsorption of TC molecules via π-π interactions, electrostatic attractions, and hydrogen bonding [47]. The incorporation of magnetic properties in MAC@NaAlg further enhances its potential due to improved accessibility and separation efficiency after adsorption [44]. Meanwhile, PBP@NaAlg demonstrated relatively good adsorption performance, which can be attributed to the functional groups present in banana peel powder (e.g., hydroxyl, carboxyl, and amine groups) that interact with TC molecules [48]. The pure sodium alginate beads (NaAlg) exhibited the lowest adsorption capacity, suggesting that the alginate matrix alone has limited active sites for TC adsorption.

The adsorption capacities of AC@NaAlg and MAC@NaAlg observed in this study are consistent with values reported for other advanced adsorbents in the literature. For example, biochar-derived adsorbents and magnetic composites have shown adsorption capacities ranging from 30 to 60 mg/g, depending on preparation methods and experimental conditions [49]. The performance of MAC@NaAlg in this study is particularly noteworthy, as it rivals other magnetic adsorbents like magnetic biochar, which are widely recognized for their efficiency in antibiotic removal from aqueous solutions [50]. Similarly, agricultural waste-based adsorbents, such as modified banana peel and corncob-derived materials, have exhibited adsorption capacities in the range of 20–50 mg/g under comparable conditions [48].

### 3.4. Isotherm Modelling

**Figure 7:**
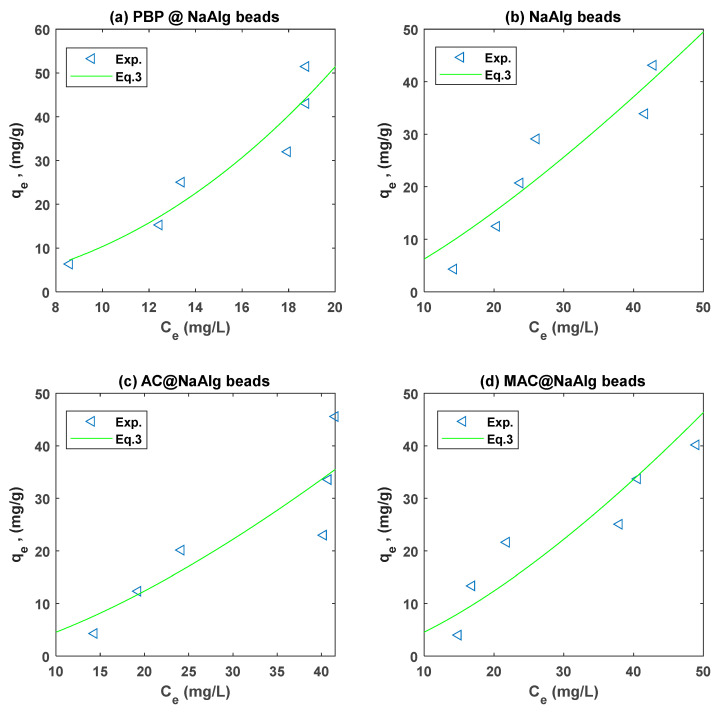
Freundlich model fit to the Cr adsorption experimental data for the different types of beads.

**Figure 8:**
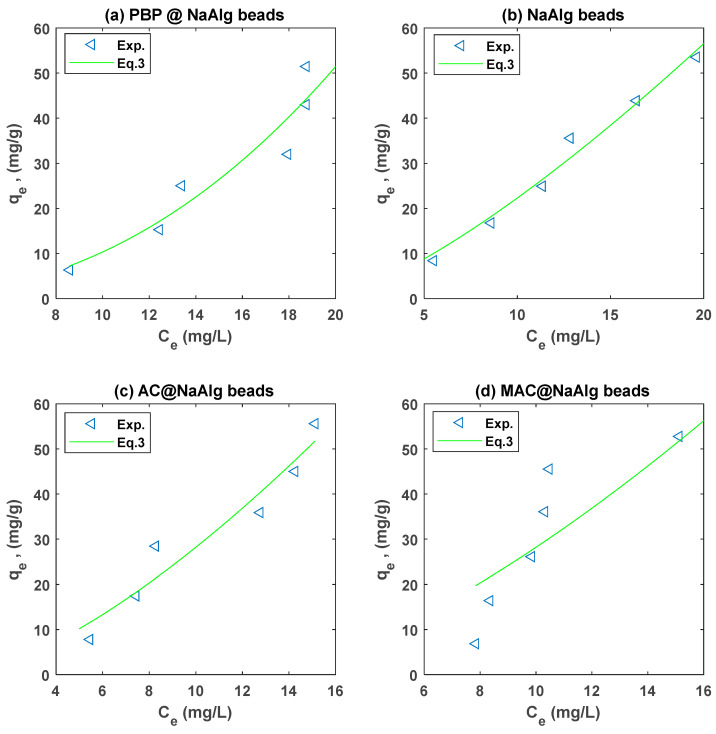
Freundlich model fit to the TC adsorption experimental data for the different types of beads.

The Langmuir and Freundlich models were fitted to the experiment on the adsorption of Cr and TC using non-linear regression. A summary of the regression parameters for the different types of beads is presented in Table 2. The Freundlich non-linear regression fits to the Cr(VI) and TC experimental data are presented in Figure 7 and Figure 8, respectively.

Table 2 presents the Langmuir and Freundlich isotherm parameters obtained from fitting experimental data for the adsorption of tetracycline (TC) and chromium (Cr(VI)) onto four different types of beads: NaAlg, PBP@NaAlg, AC@NaAlg, and MAC@NaAlg. The Freundlich isotherm parameters provide valuable insight into the adsorption performance of different bead types for Cr(VI) and tetracycline (TTC). For Cr(VI) adsorption, the PBP@NaAlg beads exhibited the highest Freundlich adsorption capacity constant ($k_{F}=3.447$) and a favourability constant n=1.299, which indicates highly favourable adsorption. This suggests that the modification of NaAlg beads with PBP significantly enhances their adsorption capacity and efficiency, which is consistent with previous studies demonstrating the effectiveness of polymer-based modifications in improving adsorption performance [47]. In contrast, NaAlg beads alone showed the lowest $k_{F}=2.207$ and an n value of 0.4321, reflecting weaker and less favourable adsorption for Cr(VI). The MAC@NaAlg beads also performed well, with $K_{f}=2.207$ and n=1.175, while the AC@NaAlg beads exhibited a moderate adsorption capacity ($K_{f}=0.0722$) and favourability (n=0.577).

For TTC adsorption, NaAlg beads showed a relatively high adsorption capacity ($K_{F}=0.8087$) with a favourable adsorption intensity (n=0.7067). The *RMSE* values were lowest for NaAlg (1.1586), indicating that the Freundlich model fits this system well. However, PBP@NaAlg beads displayed a slightly lower adsorption capacity ($K_{F}=0.4690$), though the adsorption remained favourable with n=0.7165. Interestingly, AC@NaAlg beads demonstrated a competitive adsorption capacity for TTC ($K_{F}=0.7165$) but with slightly weaker fit accuracy ($R^{2}=0.9489$ and RMSE=5.0605). MAC@NaAlg beads exhibited the lowest adsorption capacity ($K_{F}=0.2018)$, though n=0.9988 suggests very favourable adsorption. These results align with findings by [51], who noted that specific functional modifications of adsorbents could influence their selectivity and adsorption efficiency for different contaminants.

Overall, the PBP@NaAlg beads are the most effective for Cr(VI) adsorption, while NaAlg beads perform better for TTC adsorption. The differences in $k_{F}$ and n across bead types highlight the role of adsorbent modification and the nature of the adsorbate in determining adsorption performance. These results underscore the importance of tailoring adsorbent properties to target specific contaminants effectively.

### 3.5. Reuse of the Beads for Successive Adsorption

**Figure 9:**
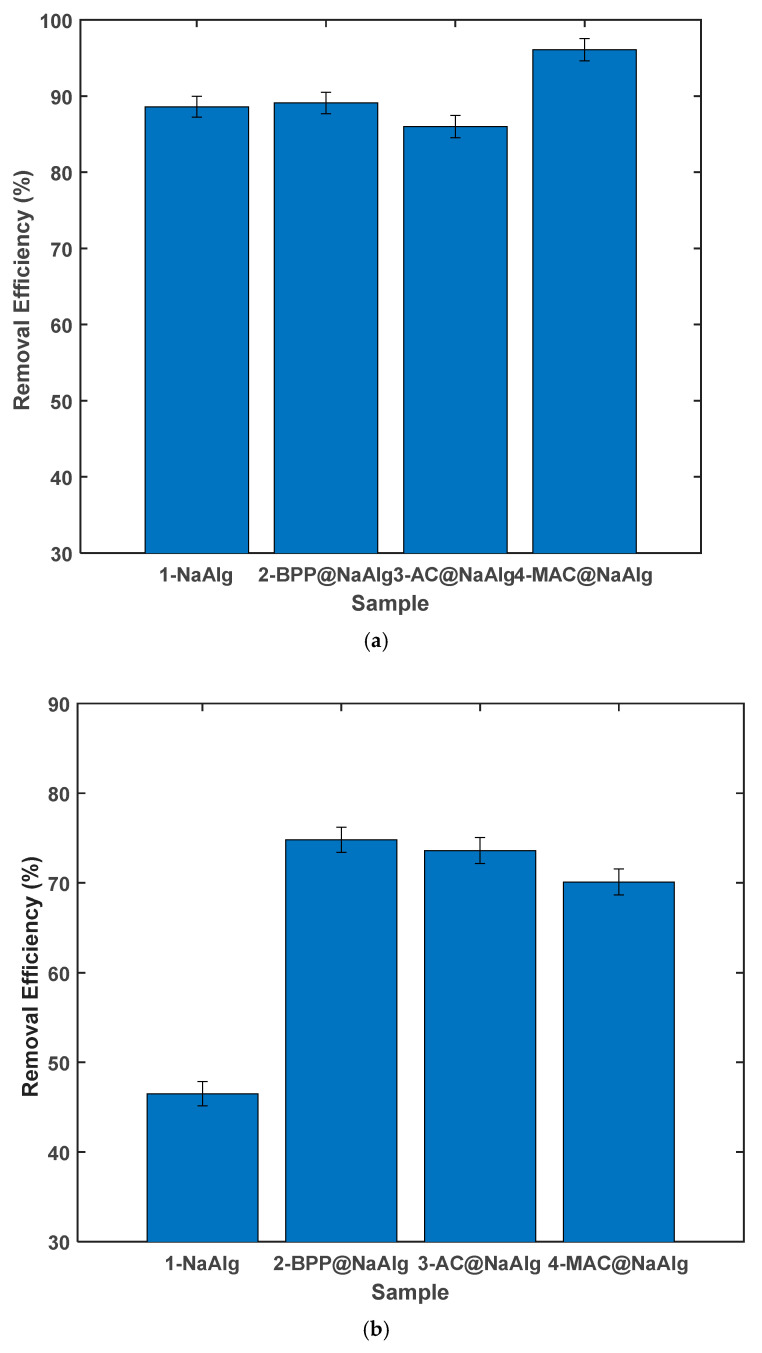
Removal efficiency of various beads in the 2nd cycle after adsorption of another pollutant (**a**) Cr(VI) followed by TC (**b**) TC followed by Cr(VI).

Figure 9 presents the results of the second cycle adsorption studies conducted with various pollutants. As observed in Figure 9a, the removal efficiency for tetracycline (TC) by the beads ranged from approximately 88% to 96%. This represents a marginal enhancement compared to the performance of the virgin beads. A similar slight improvement was also noted in the capacity of the MAC@NaAlg.

Figure 9b shows Cr(VI) removal efficiencies of the beads that have been previously used in tetracycline adsorption. The results show that the beads still had reasonable removal efficiencies ranging from 46 to 75%. However, these values were lower than those obtained in the adsorption experiments with virgin beads. Despite the beads demonstrating a remarkable capacity, one might have anticipated a decrease in their removal efficiency. This expectation is based on the assumption that the first adsorption cycle would have occupied some of the active sites and functional groups with Chromium (VI). However, the results contradict this assumption.

A plausible explanation for these findings could be that the functional groups involved in the removal of TC differ from those required for the removal of Chromium (VI). Consequently, there is no competition for active sites. Interestingly, the presence of chromium appears to enhance the removal of TC. Different pollutants might interact with different functional groups. For example, the functional groups that interact with TC might be different from those that interact with Chromium (VI). This could explain why the removal efficiency of the beads did not decrease after the first adsorption cycle: the functional groups that were occupied by Chromium (VI) in the first cycle might not be the same ones needed for the removal of TC in the second cycle.

Furthermore, the presence of chromium might have changed the chemical environment of the beads, possibly making other functional groups more accessible or effective for the removal of TC. This could be the reason why the presence of chromium seemed to enhance the removal of TC. The FTIR analysis results revealed that the adsorbent beads had the following functional groups: hydroxyl groups, carbonyl, -C-O, and C-H groups. The activated carbons can also contain the benzene ring. All these groups are known to interact with tetracycline molecules [52,53,54,55]. For example, the hydroxyl can interact with tetracycline through ionic exchange; the -NH_2_ group in the tetracycline can form a π–π interaction and a cation–π bond with the benzene ring in the samples [52].

### 3.6. Dimensional Scoring of the Different Beads

Dimensional scoring is a structured method for evaluating and comparing products based on multiple criteria, referred to as dimensions, with each representing a specific feature or attribute that is relevant to assessing a product’s performance, quality, or suitability. In this analysis, beads were evaluated using four dimensions: the removal efficiency achieved for each pollutant in the first cycle, the removal efficiency in the second cycle, the complexity of the production process, and the ease of separating the beads from the solution at the end of the process. For removal efficiency, beads were ranked numerically from 1 to 4, with 1 assigned to the bead achieving the highest efficiency. Production procedure complexity was ranked inversely, with 4 assigned to the bead with the most complex production process and 1 to the simplest. Ease of separation was ranked from 1 to 4, with 1 assigned to the bead that was easiest to separate and 4 to the most difficult. The overall score for each bead was then calculated by aggregating the ranks across all dimensions, using a specific weighting or formula to reflect their relative importance. This approach provided a comprehensive evaluation of the beads, enabling a balanced comparison across multiple criteria. The overall score was then calculated according to:

(5) $$DS=\frac{TC_{R_{eff,1}}*TC_{R_{eff,2}}*Cr{\left(VI\right)}_{R_{eff,1}}*Cr(VI)R_{eff,2}}{Prod_{effort}*Sep_{effort}}$$

Based on Equation (5), the higher the dimensional score (*DS*), the better the performance of the adsorbent.

The results from the dimensional scoring are presented in Table 3. As can be noted from Table 3, MAC@NaAlg beads had the highest score whilst NaAlg and AC@NaAlg beads had the least score of (2).

### 3.7. Comparison of Adsorption with Other Materials

Tetracycline (TC) removal from water through adsorption has been studied using various adsorbents. Graphene oxide demonstrated a high adsorption capacity (313 mg/g) and rapid kinetics, with adsorption decreasing at higher pH and Na^+^ concentrations [56]. Water treatment residues achieved 91.5% removal efficiency under optimized conditions, with an adsorption capacity of 37.194 μmol TC/g [57]. Struvite showed pH-dependent adsorption, with the lowest removal (8.4%) at pH 7.7, and approximately 22% removal during precipitation [58]. Magnetic nanocomposites exhibited excellent performance, reaching 99.8% removal efficiency at an optimal pH of 4.5–5.6 [59].

The removal capacities of the adsorbent beads produced in this work were compared with those of similar materials from the literature in Table 4 and Table 5.

Recent studies have explored various adsorbent materials for efficient Cr(VI) removal from water and soil. Magnetic alginate hybrid beads (Fe_3_O_4_@Alg-Ce) demonstrated an enhanced sorption capacity of 14.29 mg/g compared to calcium alginate and Fe_3_O_4_ particles alone [60]. Chitosan beads modified with sodium dodecyl sulphate showed a maximum adsorption capacity of 3.23 mg/g for Cr(VI) [61]. Polyvinyl alcohol-alginate beads cross-linked with boric acid and calcium chloride exhibited complete Cr(VI) removal after 1.5 h under UV light illumination [62]. A millimetre-sized polyaniline/polyvinyl alcohol/sodium alginate composite (PPS) demonstrated a high adsorption capacity (83.1 mg/g) for Cr(VI) in water and effectively removed 24.17% of the total Cr and 52.47% of the Cr(VI) from contaminated soil after 30 days [63]. These studies highlight the potential of various adsorbent materials for Cr(VI) remediation in both aqueous and soil environments.

The results from this research show that the banana peel powder-based adsorbent beads produced in this work have great potential as adsorbent materials for emerging pollutants such as antibiotics and waste materials. Its also been shown that beads still retain a reasonable adsorption capacity after the first cycle even without desorbing the first pollutant.

**Table 4:** Comparison of tetracycline removal capacity of adsorbent beads with other polymeric materials from the literature.

Material	Removal Capacity (mg/g)	Ref.
Calcium/iron-layered double hydroxides	10.393	[64]
Ionic liquid-impregnated chitosan hydrogel beads	22.42	[65]
CeO_2_@starch nano composite particles	48.54	[66]
Dye-attached polymeric microbeads	9.63	[67]
Double-network polyvinyl alcohol-copper alginate gel beads	231.43	[68]
PBP@NaAlg	51.3	This study
AC@NaAlg	51.8	This study
MAC@NaAlg	55.8	This study
NaAlg	51.9	This study

**Table 5:** Comparison of chromium (VI) removal capacity of adsorbent beads with other polymeric materials from the literature.

Material	Removal Capacity (mg/g)	Ref.
Fe_3_O_4_@Alg-Ce	14.29	[60]
Chitosan beads	3.23	[61]
Polyvinyl alcohol-alginate beads	0.17	[62]
Sodium alginate composite (PPS)	83.1	[63]
PBP@NaAlg	47.3	This study
AC@NaAlg	46.6	This study
MAC@NaAlg	42.2	This study
NaAlg	41.2	This study

### 3.8. Disposal of Used Adsorbent

Disposing of biochars loaded with metal pollutants requires careful consideration to prevent secondary contamination [69]. Some of the safe disposal methods include (1) recycling and repurposing: Metal-loaded biochars can be recycled and repurposed for various applications, such as catalytic and electrochemical materials. This approach not only safely disposes of the biochar but also recovers valuable metals [70]. (2) Secure landfills: If recycling is not feasible, the biochar can be disposed of in secure landfills designed to handle hazardous materials. These landfills have special liners and leachate collection systems to prevent pollutants from leaching into the environment. (3) Incineration: High-temperature incineration can be used to destroy organic contaminants and volatilize metals, which can then be captured and treated in air pollution control systems. (4) Stabilization and solidification: This method involves mixing the biochar with binding agents to immobilize the metals, reducing their mobility and potential environmental impact.

### 3.9. Limitations

The primary limitation of this study lies in its lack of focus on scale-up and real-world application considerations. Notably, it did not include continuous flow studies or address scale-up challenges, both of which are critical for industrial applications. Additionally, the absence of a cost analysis for the production process and an evaluation of the environmental impact of large-scale production leaves key questions about the commercial viability of this technology unanswered.

These gaps highlight essential areas for future research to enhance our understanding and optimization of banana peel-based adsorbents for practical wastewater treatment. Long-term stability over multiple cycles warrants further investigation, as does the mechanical strength of the beads. Evaluating their structural integrity is especially important for their use in packed bed columns, where mechanical durability is crucial.

## 4. Conclusions

The results show that banana peel waste, an abundant and low-cost resource, can be effectively transformed into advanced adsorbent materials, aligning with circular-economy principles and promoting waste valorization. The study demonstrates that these adsorbent beads, particularly those containing magnetic activated carbon (MAC), are highly efficient in removing pollutants such as tetracycline (up to 92%) and hexavalent chromium (up to 79%) from aqueous solutions. The characterization of the beads highlights their suitability for adsorption, with functional groups, thermal stability, and surface area contributing to their high performance. Additionally, the beads retain a significant adsorption capacity across reuse cycles without requiring the desorption of previously adsorbed pollutants, suggesting that the active sites for different pollutants, such as antibiotics and heavy metals, are distinct and non-competitive.

The research underscores the comparative advantage of banana peel-based beads over many other adsorbents reported in the literature, offering a cost-effective, sustainable, and environmentally friendly solution for wastewater treatment. Furthermore, these adsorbents show potential for broader applications, given their versatile adsorption capabilities. The study highlights the need for further research to optimize and scale-up these materials for real-world applications, making them a promising innovation for addressing water pollution while utilizing waste materials effectively.

However, several limitations and areas for future research were identified. These include the need for continuous flow studies, scale-up considerations, cost analysis, and environmental impact assessment of large-scale production. Additionally, investigation of long-term stability over multiple cycles and mechanical strength in packed bed columns would be valuable for industrial applications. Despite these limitations, this study makes a significant contribution to the field of sustainable water treatment by demonstrating the feasibility of converting banana peel waste into effective adsorbent materials, supporting both environmental remediation and waste valorization objectives.

## Figures and Tables

**Table 1:** CHNS analysis results for the banana peel powder, activated carbon beads, and magnetic activate carbon beads.

Material	Element Composition (%)			
	C	H	N	S
Banana Peel Powder	39.4	6.4	1.1	<0.3
AC@NaAlg Beads	24.7	4.5	<0.3	<0.3
MAC@NaAlg Beads	19.9	3.8	<0.3	<0.3

**Table 2:** Summary of Langmuir and Freundlich fit parameters from non-linear regression fits of the TC and Cr(VI) adsorption experimental data.

Adsorbate	Para.	Units	Bead Type			
			NaAlg	PBP @NaAlg	AC @NaAlg	MAC @NaAlg
Cr	$K_{F}$	(-)	0.0501	3.4473	0.0722	2.2068
	n	(-)	0.4321	1.2988	0.5772	1.1745
	$R^{2}$	(-)	0.8983	0.9835	0.9855	0.9620
	RMSE	(-)	6.0251	2.0927	2.6307	2.8723
TTC	$K_{F}$	(-)	0.8087	0.1667	0.2842	0.004
	n	(-)	0.7067	0.4690	0.7165	0.2018
	$R^{2}$	(-)	0.9972	0.9947	0.9498	0.9430
	RMSE	(-)	1.1586	1.4203	5.0605	3.7811

**Table 3:** Dimensional scoring of different types of beads.

Bead Type	TC Removal		Cr(VI)		Effort		DS
	1st	2nd	1st	2nd	Product	Separation	
NaAlg	3	2	1	1	1	4	2
PBP@NaAlg	3	3	4	4	2	4	18
AC@NaAlg	3	1	3	3	3	4	2
MAC@NaAlg	4	4	3	2	4	1	24

## Data Availability

The original contributions presented in this study are included in the article. Further inquiries can be directed to the corresponding author.
